# Switching Language Modes: Complementary Brain Patterns for Formulaic and Propositional Language

**DOI:** 10.1089/brain.2017.0573

**Published:** 2018-04-01

**Authors:** John J. Sidtis, Diana Van Lancker Sidtis, Vijay Dhawan, David Eidelberg

**Affiliations:** ^1^ Brain and Behavior Laboratory, The Nathan Kline Institute for Psychiatric Research, Orangeburg, New York.; ^2^ Department of Psychiatry, New York University Langone School of Medicine, New York, New York.; ^3^ Department of Communicative Sciences and Disorders, New York University Steinhardt School, New York, New York.; ^4^ Center for Neurosciences, Feinstein Institute for Medical Research, Manhasset, New York.

**Keywords:** basal ganglia, language, laterality, performance-based analysis, positron emission tomography, speech formulas

## Abstract

Language has been modeled as a rule governed behavior for generating an unlimited number of novel utterances using phonological, syntactic, and lexical processes. This view of language as essentially propositional is expanding as a contributory role of formulaic expressions (e.g., *you know, have a nice day, how are you?*) is increasingly recognized. The basic features of the functional anatomy of this language system have been described by studies of brain damage: left lateralization for propositional language and greater right lateralization and basal ganglia involvement for formulaic expressions. Positron emission tomography (PET) studies of cerebral blood flow (CBF) have established a cortical–subcortical pattern of brain activity predictive of syllable rate during phonological/lexical repetition. The same analytic approach was applied to analyzing brain images obtained during spontaneous monologues. Sixteen normal, right-handed, native English speakers underwent PET scanning during several language tasks. Speech rate for the repetition of phonological/lexical items was predicted by increased CBF in the left inferior frontal region and decreased CBF in the head of the right caudate nucleus, replicating previous results. A complementary cortical–subcortical pattern (CBF increased in the right inferior frontal region and decreased in the left caudate) was predictive of the use of speech formulas during monologue speech. The use of propositional language during the monologues was associated with strong left lateralization (increased CBF at the left inferior frontal region and decreased CBF at the right inferior frontal region). Normal communication involves the integration of two language modes, formulaic and novel, that have different neural substrates.

## Introduction

Connectivity has become a significant component of imaging approaches to describing the functional anatomy of many behaviors. Although speech and language are highly complex behaviors that are not fully understood on their own, they do have a potential advantage with respect to brain mapping. The lateralization of speech and language to the left cerebral hemisphere in right-handed individuals has been a cornerstone of neurological localization since the 19th century. This functional organization has been reliably demonstrated by repeated clinical observations after unilateral brain damage (Davis and Wada, 1978). However, achieving refinement in our understanding of the neurology of spoken expression has proven especially challenging using functional imaging approaches.

An important consideration in mapping speech and language is that these behaviors are expressed in different modes during normal communication. Language is generally characterized as propositional with phonologically based morphological and lexical items ordered by grammatical rules to express an idea (Chomsky, 1957). This is the mode of language that can be devastated by left hemisphere damage, resulting in a form of aphasia depending on the location of the lesion. Less well recognized, however, is the fact that many aphasic individuals retain an expressive ability, originally referred to as automatic speech (Hughlings Jackson, 1932; Van Lancker Sidtis, 2012). Hughlings Jackson's (1932, p. 183) cited examples, such as *take care, that's a lie, goodbye, oh dear, bless my life,* typify the modern conception of formulaic language. Prominent aphasiologists in the 20th century invariably mentioned the dramatic contrast between these preserved, well-articulated unitary utterances and disordered, newly created speech (Alajouanine, 1956; Bay, 1964; Blanken, 1991; Code, 1982; Critchley, 1970; Espir and Rose, 1970; Gloning et al., 1963; Goldstein, 1948; Goodglass and Mayer, 1958; Head, 1926; Luria, 1966; Pick, 1931; Wepman et al., 1956). The preserved ability to produce properly articulated and intoned utterances such as *hello; see you later; I came, I saw, I conquered; two wrongs don't make a right,* was generally viewed as a curiosity. Such utterances, which are exceedingly numerous in normal discourse (Jackendoff, 1995), are now recognized as constituting formulaic language (Wray and Perkins, 2000), which makes up a considerable portion, about one-fourth, of conversational interaction (Van Lancker and Rallon, 2004).

In contrast to propositional (novel, grammatical) language, which is rule based and generates newly created utterances, formulaic language consists of a large number of fixed expressions with unique characteristics (Wray, 2002), which are stored and processed as coherent, stereotyped forms with conventionalized meanings (Kuiper, 2000; Lin, 2010). Formulaic expressions differ from novel speech in important ways. They are stored as canonical forms with specific words in a certain order and a signature phonetic and intonation pattern (Rammell et al., 2017). Word meanings are based on nonliteral strategies and do not represent the usual lexical semantics, nor are grammatical forms always honored. Formulaic expressions are usually highly nuanced (have strong connotative content) with negative or positive or valence, as in *he's at the end of his rope*, *no sooner said than done*, and *there's going to be hell to pay*. For example, *he's out on a limb* conveys tension and anxiety, whereas a comparable literal expression *he's out on a boat*, without further information, is relatively neutral with respect to nuance. Functionally, formulaic expressions are used to achieve bonding, humor, and group affiliation and to maintain fluency (Wray and Perkins, 2000).

Propositional language and formulaic language differ not only in linguistic form and function but in their neurological organization as well. The proportions of formulaic expressions in spontaneous speech are significantly increased after left hemisphere damage and aphasia (Van Lancker Sidtis and Postman, 2006; Yang and Van Lancker Sidtis, 2016). In contrast, a pathological diminution of formulaic expressions occurs in right hemisphere (Van Lancker Sidtis and Postman, 2006) or subcortical damage from stroke (Sidtis et al., 2009; Speedie et al., 1993; Van Lancker Sidtis et al., 2016) or Parkinson's disease (Bridges et al., 2013; Illes, 1989; Van Lancker Sidtis et al., 2016). Abundance of formulaic language in Alzheimer's disease, in which subcortical nuclei remain functional until late in the disease, further supports the role of the basal ganglia in formulaic production (Bridges and Van Lancker Sidtis, 2013), likely reliant on procedural processes of those structures (Ullman, 2004), drawing on habitual or procedural memory (Mishkin et al., 1984). These studies of persons with left or right hemisphere damage or subcortical impairments form the foundation of the dual process model of language (Erman and Warren, 2000; Nespoulous et al., 1998; Wray and Perkins, 2000): propositional, novel, newly created language is represented in the left hemisphere, whereas formulaic (overlearned, routinized) language is modulated by a right hemisphere–subcortical system (Heine et al., 2014; Van Lancker Sidtis, 2012).

We previously identified a straightforward, clinically relevant, reliable pattern of blood flow changes in the brain with positron emission tomography (PET) that is predictive of speech rates in normal subjects (Sidtis, 2012; Sidtis et al., 2003) and individuals with hereditary spinocerebellar ataxia (Sidtis et al., 2006, 2010). The performance-based analysis used a stepwise multiple linear regression analysis to determine whether a linear combination of regional blood flow values (independent variables) could significantly predict speech rate in syllables per second (dependent variable) during scanning. A significant linear regression solution representing an inverse blood flow relationship between the left inferior frontal region of the cerebral cortex and the head of the right caudate nucleus of the basal ganglia predicted speech rate during the repetition of phonological items. Left frontal blood flow increases and right caudate blood flow decreases as speech rates increase. This relationship was not observed in the group mean data, which tended to be symmetrical with respect to laterality, nor in task contrasts (speech–rest), which tended to produce an uninterpretable laterality (Sidtis, 2007). The involvement of these brain areas in speech is consistent with clinical observations of language disorders (Caplan et al., 1990; Geschwind, 1970), with the left inferior frontal area classically associated with expressive propositional language and the right caudate associated with normal speech production.

In this study, we used the performance-based analysis to compare cortical–subcortical interactions during the repetition of phonological and lexical items with the production of words in propositional and formulaic utterances. The aim of this study is to develop a better understanding of cortical–subcortical interactions during different speech and language modes as a foundation for an accurate mapping of the functional anatomy of spoken expression.

## Materials and Methods

### Subjects

For this study, a total of 128 whole-brain cerebral blood flow (CBF) scans were obtained from the 16 normal subjects. There were nine females and seven males with a mean age of 57 ± 10 years. All subjects were right-handed, native speakers of American English. None had a history of diagnosed neurological or psychiatric disease. None were taking psychotropic medication and none had abnormal speech. All PET scans were performed at the Feinstein Research Institute of North Shore-Long Island Jewish Medical Center in accordance with the protocol approved by their Institutional Review Board. Speech studies were conducted in accordance with the protocol approved by the Nathan Kline Institute/Rockland Psychiatric Center Institutional Review Board. All subjects provided informed consents for both the speech and PET components of this study.

### PET imaging procedures

Subjects generally arrived at the PET suite at 8:00 am to be consented, interviewed, and instructed in the procedures. Subjects were then positioned in the PET scanner (GE Advance Tomograph, General Electrics) and an intravenous line was placed in the subject's left arm for H_2_^15^O injection at ∼10:00 am. A stereotactic head holder and 3D laser alignment were used for stable and reproducible head positioning. Lightweight headphones were attached to the head holder to facilitate communication with the subject. A 10 min transmission scan was performed for attenuation correction followed by a 2D PET scan to establish the delay time between H_2_^15^O injection and the detection of brain activity by the scanner. This was followed by a series of whole-brain 3D PET scans, with two scans for each of the four speech tasks (three syllable and word repetition tasks, one monologue task). Based on the observed brain delay time, each speech task was initiated 15 sec before detection of H_2_^15^O in the brain. Tasks were performed for 60 sec using the procedure reported previously (Sidtis, 2012, 2015; Sidtis et al., 2003, 2006, 2010). Blood flow was measured using a modified slow bolus injection of H_2_^15^O using an automated injection system and image acquisition lasting ∼2 min.

### PET image processing

Scans were reconstructed using the 3D reprojection (3D RP) method, matrix dimensions 128 × 128 × 35, with voxel dimensions of 2.34 × 2.34 × 4.25 mm, with no smoothing applied. PET images were first aligned within subject and then spatially normalized to a standard space using the SPM99 software (SPM, London, United Kingdom; www.fil.ion.ucl.ac.uk/spm/). Regions of interest used in previous PET-speech studies (Sidtis, 2012; Sidtis et al., 2003, 2006, 2010) extracted multiple regional CBF values from the ventral to dorsal extent of the head of the caudate, and regional values from the ventral to dorsal extent of the inferior frontal regions, bilaterally, using ScanVP image analysis software (Spetsieris et al., 1993). Irregular regions were used and adjusted on an individual basis to ensure capture of the target structure. However, regions were constant within a subject across all speech conditions. A threshold was applied to each region so that the upper 10% of activity was captured to reduce partial volume errors and to minimize individual differences in anatomy. For each scan, a global CBF value was obtained using a whole-brain region of interest. This was used for normalization across subjects. Neither the data for the repetition nor the monologue scans were contrasted with the data for any other scanning conditions.

### Speech samples

Four speech tasks were used: repeated productions of the syllable/*pa*/, the syllable sequence/pa-ta-ka/, the sentence/pop-the-top-cop/, and a spontaneous monologue on a topic of the subject's choice. All monologues were generated by the speakers without control or direction by the examiners. Therefore, each instance of a monologue was unique. Monologues consisted primarily of propositional (novel, grammatical, newly created) utterances, interspersed with unitary, formulaic expressions. Examples of novel and formulaic expressions taken from participants' monologues appear as follows:

Novel examples:
*We went with another couple.*
*I walked back to the house.*
*In aerobics we danced 18 hours a week.*

Formulaic examples:
*On the face of the earth.*
*She could talk a mile a minute.*
*Vanishes in a puff of smoke.*

Each task was performed twice, each occurrence lasted 60 sec, and each was associated with a PET scan. These tasks were performed in random order in the first half of the study and then repeated in reverse order in the second half. The speech samples used to extract dependent measures for the performance-based analysis were digitally recorded during scanning. Syllable rates were measured during the syllable and word production tasks, and the total number of words, the number of words in formulaic expressions, and the number of words in propositional expressions were determined for each spontaneous monologue. Syllable rate refers to the number of syllables per second produced by each speaker during the 60-sec production period. All of the speech produced during scanning was digitally recorded. The number of syllables produced during the 60-sec periods for the repetition of the syllable/pa/, the syllable sequence/pa-ta-ka/, and the word sequence/pop-the-top-cop/ was counted to compute the syllable rates. These speech samples comprise syllables and words, which are the building blocks of novel language, and in this study, they are used as a surrogate, in the experimental setting, of propositional language. The total number of words and the number of words in the propositional and formulaic expressions were computed for the monologue productions to determine the proportion of words in each type of expression.

Identification of formulaic expressions was accomplished, using form and function criteria established previously, by two independent raters, who achieved consensus regarding any discrepancy (Van Lancker and Rallon, 2004). Raters were native speakers of American English who were trained in the identification and analysis of formulaic expressions. Categories of formulaic expressions were derived originally from observations in persons with left hemisphere damage and aphasia. In our research, formulaic expressions are identified, first, using native speaker intuition and second, by applying formal and functional criteria, described more fully in Van Lancker and Rallon (2004). Native speakers are aware when they “know” an expression, such as an idiom, proverb, or conversational speech formula. Formal criteria include cohesion in the words comprising the expression and use of nonstandard semantics and grammar. Functionally, formulaic expressions contribute to fluency, social bonding, and turn taking. In our studies, we focus on speech formulas (e.g., *how are you*), discourse elements (*like, ya know*), conventional expressions (*in the meantime, as far as I know*); idioms and proverbs; nonlexical pause fillers (*uh, um*), and utterance initials (*I think, I guess*). All speech samples were digitally recorded during scanning for analyses. Recordings were made using a primary and back-up Marantz Professional digital recorders (PMD660) with boom-mounted Audio-Technica AT3035 (primary) and AKG D5 (secondary) microphones. All recordings were made in .wav format at a 48k sampling rate.

### Statistical analysis

For the performance-based analysis of speech rate and formulaic language use, the regional CBF data were normalized using the ratio between the highest whole-brain CBF value in the data set and the global CBF value for the scan from which the regional values were measured (Sidtis, 2012, 2015; Sidtis et al., 2003, 2006, 2010). The globally normalized CBF data from the left and right heads of the caudate nuclei and inferior frontal regions for each of the repetition task scans were used as predictor variables for the repetition rate measured during each scan (outcome variable) in a stepwise multiple linear regression analysis (SPSS for PC version 7.5). The performance-based analysis uses the stepwise multiple linear regression to determine whether there is a linear combination of regional CBF data that predicts a performance measure such as repetition rate or vocal stability (Sidtis, 2012, 2015). This statistical procedure assesses the contribution of each potential predictive region to establishing a significant linear relationship with the dependent variable. Variables are entered into a regression model, tested, and either retained or rejected. The following criteria were used for all regression analyses: probability of *F* to enter (0.05), probability of *F* to remove (0.10), and tolerance (0.01). Although over-fitting and under-fitting regression models can be a concern with this approach, cross-validation is recommended as a confirmatory procedure. The prediction of speech rate provided a cross-validation of the stepwise multiple regression analysis with previous functional imaging studies by replicating their results (Sidtis, 2012; Sidtis et al., 2003, 2006, 2010). Moreover, the brain regions identified using the stepwise multiple liner regression replicated the effects of brain lesions to these areas in clinical studies, supporting the validity of the analysis.

Comparable analyses were performed for the proportion of words used in propositional utterances, and the proportion of words used in conversational formulaic expressions. These proportions were computed for each monologue and were used as the dependent measures for separate stepwise multiple regressions using the caudate and inferior frontal CBF data as potential predictors. As the number of scans available for these analyses were one-third the number available for the phonological–lexical repetition analyses, two-step multiple linear regressions were used. The first step (block) identified an inferior frontal region associated with the proportion of words using a step-wise multiple linear regression. The second step started with the associated inferior frontal region using the enter procedure and then examined a possible role for the caudate using a stepwise procedure. The same criteria for variable inclusion and exclusion already described were used in both stages.

## Results

The acoustic analysis revealed and average speech rate of 4.1 ± 0.8 (mean ± standard deviation) syllables per second across the three phonological/lexical repetition tasks. For the monologues, there was an average of 157.7 ± 36.1 words produced. Of these, 3.3% ± 3.0% occurred in conversational speech formulas. The propositional expressions represented 92.8% ± 4.0% of the words produced in the monologues.

**FIG. 1.**
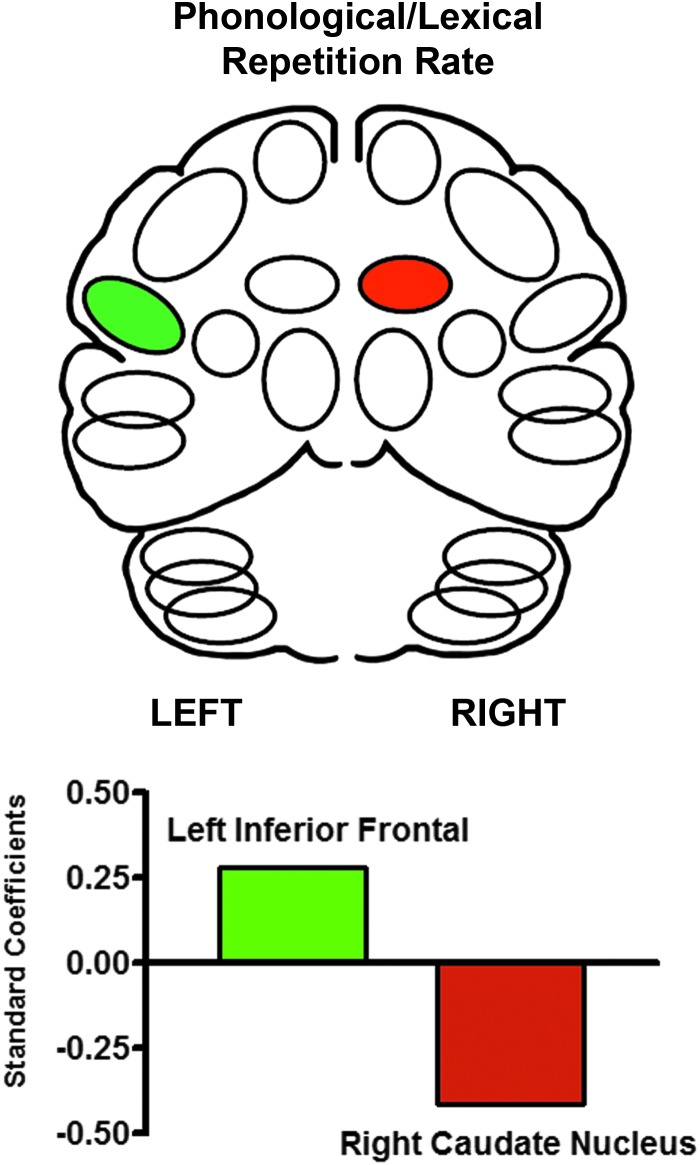
The significant multiple linear progression predictors of speech rate during the repetition of phonological and lexical items. The standardized beta regression weight for left inferior frontal blood flow (+0.28) increases while the regression weights for the right caudate nucleus decrease (−0.41) as the rate of syllable production increases. This predictive pattern has been previously reported for normal and ataxic speakers.

Multiple linear regression (stepwise) that included the left-right pairs of inferior frontal and caudate regions determined a significant predictive pattern for speech rate for the phonological and lexical repetition tasks [*F*(2, 89) = 5.09; *p* < 0.001]. The model consisted of a negative standardized beta weight (−0.41) for a right caudate region and a positive standardized beta weight (+0.28) for a left inferior frontal region (Fig. 1). This pattern replicated the predictive models previously published for normal subjects and those with hereditary ataxia (Sidtis, 2012; Sidtis et al., 2003, 2006, 2010).

**FIG. 2.**
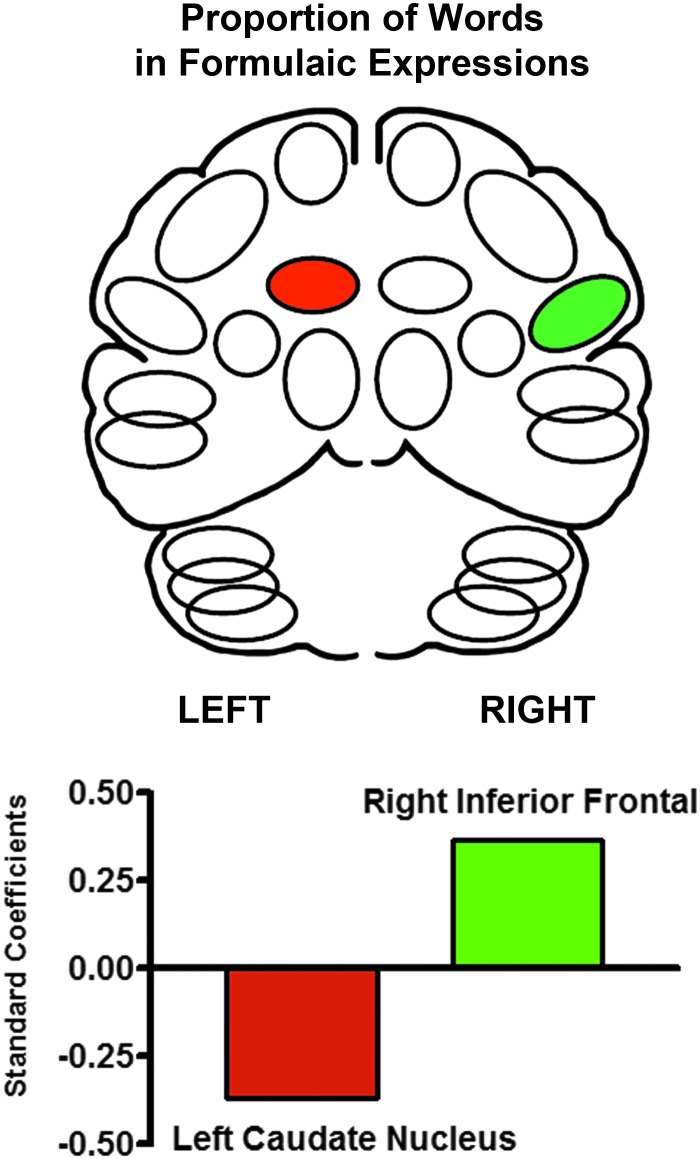
The significant multiple linear progression predictors of formulaic expression use during spontaneous monologues. The standardized beta regression weight for left caudate blood flow was negative (−0.37), whereas the regression weights for the right inferior frontal region (+0.37) were positive. This pattern is complementary to the pattern found for syllable rate during the repetition of phonological and lexical items.

A comparable analysis was applied to predicting the proportion of words in formulaic expressions in the monologues. A significant predictive pattern was found for the proportion of words in formulaic expressions in the monologues [*F*(2, 29) = 7.45; *p* = 0.002] consisting of a positive standardized beta weight for a right inferior frontal region (+0.37) and a negative standardized beta weight for a left caudate region (−0.37; Fig. 2).

**FIG. 3.**
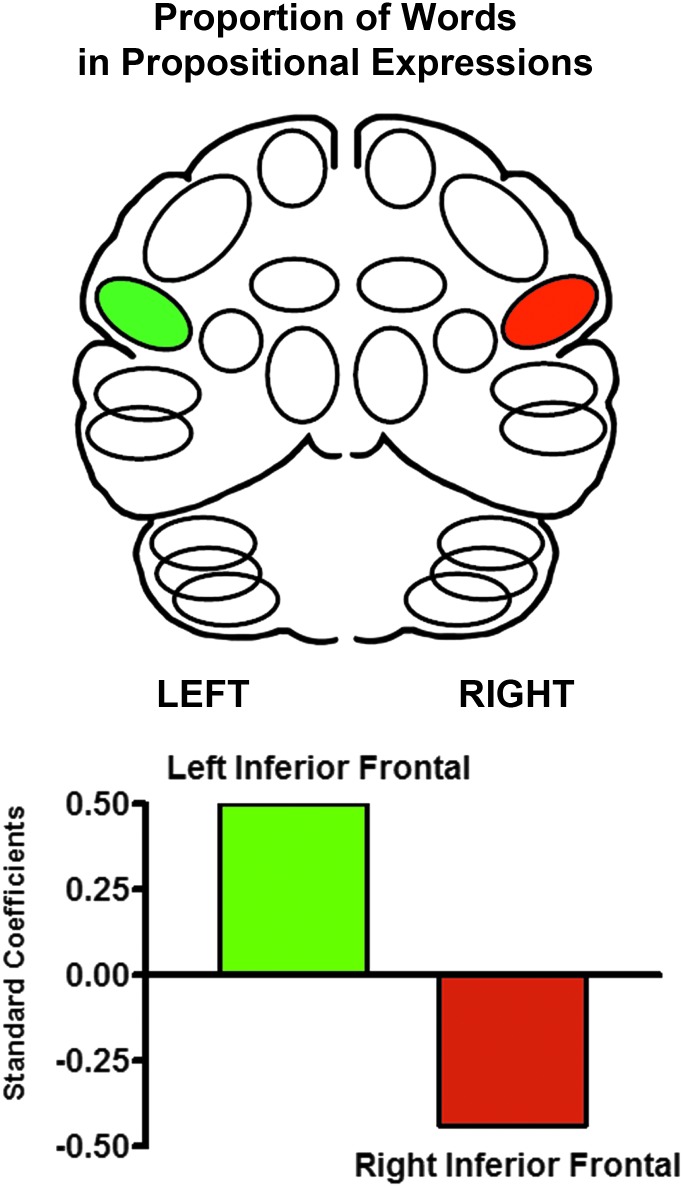
The significant multiple linear progression predictors of the percentage of words in propositional expressions during spontaneous monologues. The standardized beta regression weights for left inferior frontal blood flow were positive, (+0.51) whereas the regression weights for the right inferior frontal blood flow (−0.44) were negative. This pattern represents the strong cerebral functional asymmetry for speech and language that is found after unilateral brain injury in right-handed individuals.

This analysis was repeated for the proportion of words in propositional expressions. A significant predictive pattern [*F*(2, 29) = 7.86; *p* = 0.002] was also found. This pattern consisted of left and right inferior frontal regions with a positive standardized beta weight for a left inferior frontal region (+0.51) and a negative standardized beta weight for a right inferior frontal region (−0.44) (Fig. 3).

## Discussion

The results of the phonological/lexical repetition task replicate the inverse relationship between CBF in the left inferior frontal region and the head of the right caudate nucleus associated with speech rate previously reported in normal and ataxic speakers (Sidtis, 2012; Sidtis et al., 2003, 2006, 2010). The results also demonstrated a complementary pattern of laterality (increased CBF in the right inferior frontal region and decreased CBF in the head of the left caudate nucleus) associated with the proportion of words in formulaic expressions during a monologue. As with the phonological/lexical repetition result, the complementary laterality pattern of results for the use of speech formulas is consistent with studies of individuals with neurological disorders. The pattern of CBF associated with the proportion of words in propositional speech did not have a cortical–subcortical relationship, but it did emphasize the left-dominant brain laterality (left inferior frontal increase and right inferior frontal decrease) for propositional expression reported in clinical studies since the mid-19th century.

The repeated observation that the caudate plays a role in motor speech control is consistent with reports on the involvement of this structure in fluency. Disordered speech can occur after damage to either the left or the right caudate, but this abnormality may be more common following right-sided lesions (Caplan et al., 1990). Greater speaking ability in aphasic individuals has been associated with higher relative glucose metabolism in the left caudate, whereas poorer speaking ability was associated with higher relative glucose metabolism in the right caudate (Metter et al., 1984). In progressive hereditary spinocerebellar ataxia, higher right caudate blood flow was associated with more severe dysarthria (Sidtis et al., 2006). Furthermore, stuttering in children has been associated with an abnormally small right caudate (Foundas et al., 2013). Abnormal basal ganglia function has been implicated in stuttering (Alm, 2004), but the nature of the dysfunction remains unclear. In normal bilingual speakers, the caudate also appears to play a role in controlling the language in use (Crinion et al., 2006). It has been suggested that the involvement of the right caudate in the left hemisphere process of speaking suggests an inhibitory rather than facilitatory role for this process (Sidtis et al., 2006). Conversely, the involvement of the left caudate in a complementary manner with the right inferior frontal region associated with formulaic expression suggests that both left and right caudate nuclei reflect inhibitory processes to support unilateral control during specific linguistic modes. This is consistent with the basal ganglia's role in selecting one motor program and inhibiting others (Mink, 1996), a component of planning, initiation, and stopping motor activities (Aron and Poldrack, 2006; Graybiel et al., 1994).

The complementary patterns of cortical–subcortical interactions found for the repetition of phonological/lexical items and the use of formulaic expressions reflect the clinical observations mentioned previously, whereby persons with left hemisphere damage produce a significantly higher proportion of formulaic expressions in monologue speech, whereas right hemisphere or subcortical damage or dysfunction is associated with significantly lower proportions when compared with healthy speakers. These observations lead to the dual process model of language representation. Novel, grammatical production of propositional language is modulated by the left hemisphere, whereas formulaic expressions are produced with significant contributions from the right hemisphere and subcortical nuclei. A previous study using PET imaging identified counting from 1 to 10 with activation sites in the basal ganglia, whereas naming was associated with blood flow in the left hemisphere (Van Lancker et al., 2003). With respect to the right hemisphere, several decades of neuropsychological research suggest its preferences for longer (linguistic) segments and unitary material, sensitivity to social context, and emotional experiencing. These characteristics provide a hospitable substrate for the large repertory of holistic, affectively nuanced, and context-dependent formulaic expressions. As noted previously, the basal ganglia contribute to initiating and monitoring complex motor gestures. This competence also forms a logical basis for facilitating the production of formulaic expressions, when viewed as overlearned, holistically produced (verbal) gestures (Graybiel, 1998).

Normal conversational language consists of a highly coordinated mixture of novel and formulaic modes, which are utilized to exchange and communicate ideas. These modes, at the neurological level, can be described as the product of dynamically switching neurological systems. This study provides functional imaging support for this position. Functional imaging research on language began with studying specific speech and linguistic tasks, but more recently has begun to consider widespread functional connectivity across the brain. A recent analysis of functional magnetic resonance imaging activation data from multiple studies using graph theory identified several networks involved in speech that emerged from the prefrontal cortex, insula, putamen, and thalamus (Fuertinger et al., 2015). Although provocative, this approach, like earlier task-oriented studies, did not capture the clinical experience of strong left hemisphere lateralization of speech control. Task-oriented functional imaging studies also previously examined propositional and nonpropositional speech (e.g., counting, reciting days of the week) (Larsen et al., 1978). Unfortunately, these and other studies also failed to replicate the hemispheric lateralization of these different language modes observed in clinical studies, yielding bilateral changes in CBF for both propositional and nonpropositional speech (Blank et al., 2002; Bookheimer et al., 2000). The frequent discrepancies between clinical observations and functional imaging results led to the development of performance-based analysis, which evaluates functional imaging data with respect to performance measured during scanning rather than by simply identifying brain areas where the image signal increases (Sidtis, 2012). Analyzed in this way, functional imaging results have more closely corresponded to the clinical experience.

## Conclusions

The cortical–subcortical interactions described in this study represent minimal networks, in contrast with the extensive connectivity across brain structures observed with white matter imaging and with functional connectivity based on temporally correlated changes in blood oxygen level-dependent MRI signals. However, the simple networks identified in this study are based on highly reproducible relationships derived from actual performance during scanning. They are not dependent on assumptions about task or image decomposition, and, importantly, are sensitive to individual differences. These results form part of our ongoing effort to understand cortical–subcortical relationships as a function of language tasks. There is no attempt to represent all possible connections between cortical and subcortical regions. This is a starting point for the process of describing complex, performance-based brain networks. Most importantly, the cortical–subcortical interactions for each of the speech and language tasks examined are convergent with long-standing clinical observations: the left cerebral hemisphere is dominant for propositional language in right-handed individuals, whereas the right hemisphere and basal ganglia play a significant role in the production of formulaic expressions. The clinical relationships are not trivial, as they provide a tangible foundation for describing brain–behavior relationships. Furthermore, the present results provide functional support for new treatment approaches in language disorders (Stahl and Van Lancker Sidtis, 2016). Like other complex behaviors, mapping speech and language in the brain will not identify a single network, however complex, but will require characterizing multiple constituent and interacting neurological systems that have direct relationships with behavior.
